# Therapeutic Efficacy and Cost Effectiveness of High Cut-Off Dialyzers Compared to Conventional Dialysis in Patients with Cast Nephropathy

**DOI:** 10.1371/journal.pone.0159942

**Published:** 2016-07-28

**Authors:** Adriano Curti, Albin Schwarz, Johannes Trachsler, Yuki Tomonaga, Patrice M. Ambühl

**Affiliations:** 1 Renal Division, Stadtspital Waid Zurich, Zurich, Switzerland; 2 Epidemiology, Biostatistics and Prevention Institute, Medical Economics, University of Zurich, Zurich, Switzerland; Postgraduate Medical Institute, INDIA

## Abstract

**Background:**

High Cut-Off (HCO) dialysis membranes efficiently reduce serum free light chain (FLC) concentrations and may improve renal recovery and survival from multiple myeloma (MM) associated renal failure with cast nephropathy. However, clinical trials comparing dialysis with HCO versus conventional filters are lacking. The aim of this study was to assess clinical outcomes and economic impact of HCO dialyzers compared to conventional hemodialysis membranes in cast nephropathy.

**Methods:**

Multicenter retrospective analysis of 19 patients treated for renal failure from FLC associated cast nephropathy with standard induction chemotherapy (bortezomib/dexamethasone). We compared hemodialysis treatment with High Cut-Off (n = 12) versus conventional dialyzers (n = 7). Primary endpoint was survival; secondary endpoints were renal recovery, renal function and treatment costs.

**Results:**

At 12 months, patient survival was 25% in the HCO group versus 0% in controls (p = NS). A tendency towards faster renal recovery (p = 0.066) and better renal function at 3, 6 and 12 months (p = 0.109) after diagnosis of MM was noted in the HCO group. Complete renal response rate was achieved in 10.5 and 0% of HCO and control patients, respectively, partial renal response in 15.8 and 5.3%, and minor renal response in 26.3 and 15.8%, respectively. Both patient survival and renal recovery were significantly correlated with the extent of free light chain (FLC) reduction in serum. Median treatment costs were CHF 230’000 and 223’000 (p = NS) in the HCO and control group, respectively.

**Conclusions:**

Hemodialysis treatment with HCO membranes for cast nephropathy tended towards better survival as well as faster and better recovery of renal function versus conventional dialyzers. Moreover, total medical costs were comparable between groups. In the absence of results from randomized prospective trials on this topic, the use of HCO dialyzers in patients with renal failure from cast nephropathy may be recommended. Prospective randomized trials are required.

## Introduction

Around 20% of patients with MM initially present with renal impairment and approximately 50% develop renal failure in the course of their disease. Kidney injury was present in nearly 50% of patients with MM at first presentation in a cohort of 1027 consecutive patients with newly diagnosed MM from 1985–1998 [1]. More recently, about 20% presented with renal failure, and less than 10% required renal replacement [2–4]. Cast nephropathy [5] is induced by free light chains (FLCs) produced by plasma cells and precipitating in the distal tubule, thus forming insoluble aggregates and casts, which eventually may lead to renal failure [6].

Renal failure reflects advanced disease and high tumor burden and limits therapeutic options due to related toxicities. Moreover, patients with renal failure require more and longer hospitalizations. In the setting of dialysis dependent kidney failure survival was only a few months in the past, and reversal of renal failure was a better prognostic factor than chemotherapy response [2, 7]. Recently, the outcome of MM could be improved largely by novel chemotherapeutic agents such as proteasome inhibitors (i.e. bortezomib or carfilzomib) and immunomodulatory agents (i.e. thalidomide or lenalidomide) and autologous stem cell transplantation (ASCT) [8, 9]. Several strategies to diminish FLCs have been tried in the last twenty years. Plasmapheresis was not beneficial in three RCTs [10–13]. In 2006, Hutchinson et al. were the first to demonstrate efficient elimination of both kappa and lambda FLCs by a protein leaking dialyzer [13–15]. Combination of extended hemodialysis by High Cut-Off dialyzers (HCO) and chemotherapy with bortezomib/thalidomide/high dose dexamethasone results in renal recovery in 63% of patients [16]. HCO dialyzers selectively remove FLCs from serum and, in combination with a chemotherapy consisting of bortezomib and dexamethasone, efficiently reduce FLC concentrations, resulting in prolonged freedom from dialysis [17, 18]. However, clinical trials which directly compare HCO dialyzers with conventional dialysis in patients with cast nephropathy are lacking. Therefore, the aim of this study was to assess clinical outcomes and economic impact of treatment with HCO dialyzers compared to conventional hemodialysis membranes in patients with cast nephropathy.

## Subject and Methods

Multicenter retrospective analysis in patients treated for renal failure from FLC associated cast nephropathy between July 2005 and April 2014. Eight medical centers in Switzerland participated in this study (listed under Acknowledgments). Inclusion criteria were:

- Clinical and laboratory evidence of multiple myeloma as defined by the criteria of Durie and Salmon [19]
- Biopsy proven cast nephropathy
- Renal failure with eGFR < 15 ml/min/1.73m^2^ at initial presentation or requiring renal replacement therapy
- High dose dexamethasone and bortezomib as first line induction chemotherapy
- No plasmapheresis during treatment

**Fig 1:**
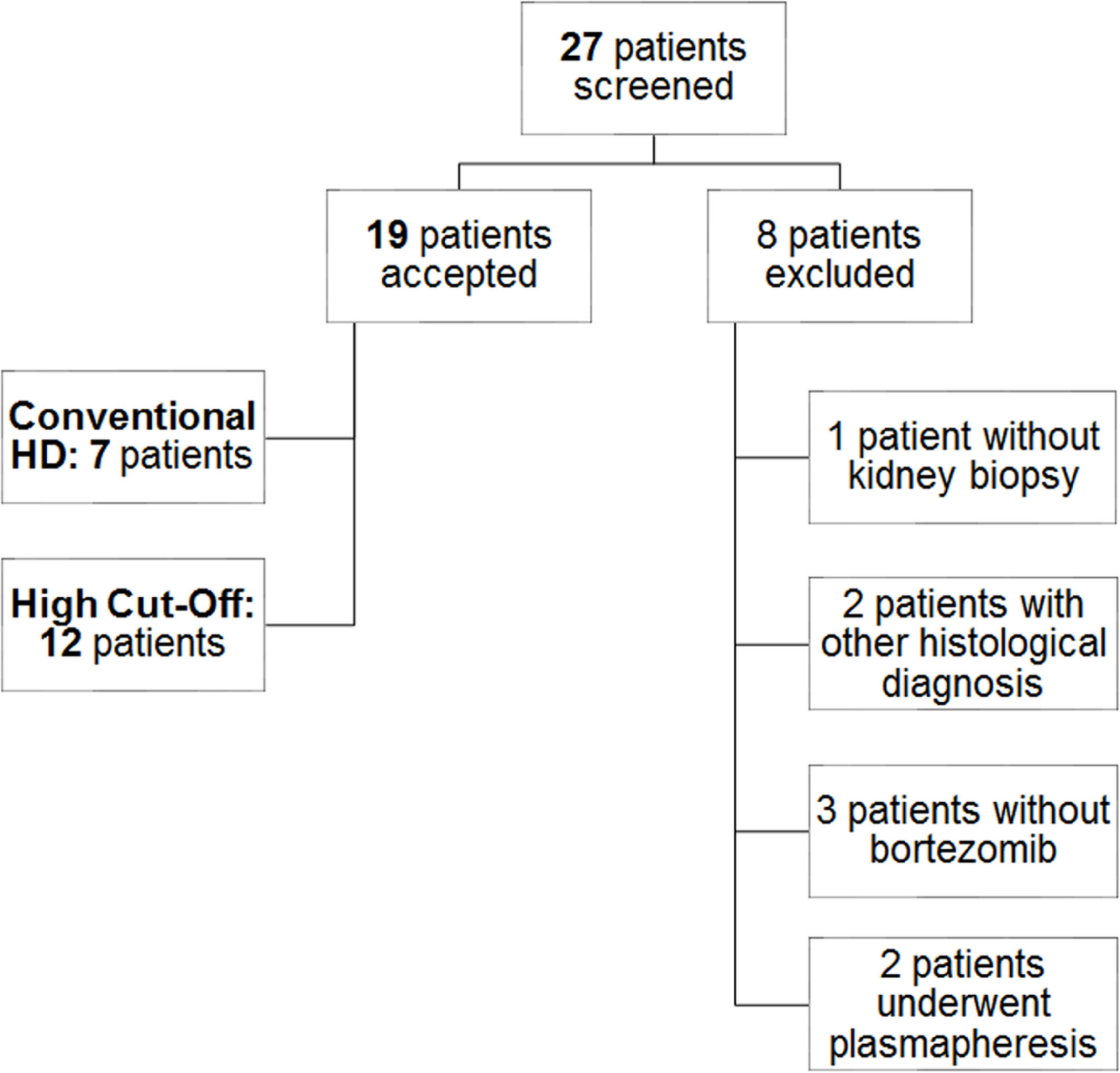
Patient screening and selection chart.

Of 27 screened individuals, 19 patients met inclusion criteria (Fig 1), 14 with new onset MM and five with relapsing disease. One patient was detected to have large FLC complexes, when treatment with HCO dialyzers had failed to reduce FLC concentrations. Diagnosis of cast nephropathy was defined by presence of at least three casts per kidney section, with typical fractured and polychromatophilic appearance by light microscopy, presence of giant cell reaction around the casts and positive staining of the casts by immunofluorescence with anti-κ or anti-λ conjugate antibodies. The degree of restoration of renal function was defined according to the criteria of the International Myeloma Working Group (IMWG) [20]: partial renal response (PR_renal_) with sustained improvement of CrCl/eGFR from < 15 ml/min at baseline to 30–59 ml/min; minor renal response (MR_renal_) with sustained improvement of baseline CrCl/eGFR from < 15 mL/min to 15–29 mL/min., or, in case of baseline CrCl/eGFR between 15–29 mL/min with improvement to 30–59 mL/min. Complete renal response (CR_renal_) is defined as the improvement of CrCl/eGFR from < 50 ml/min at baseline to > 60 ml/min persisting for at least 2 months.

**Fig 2:**
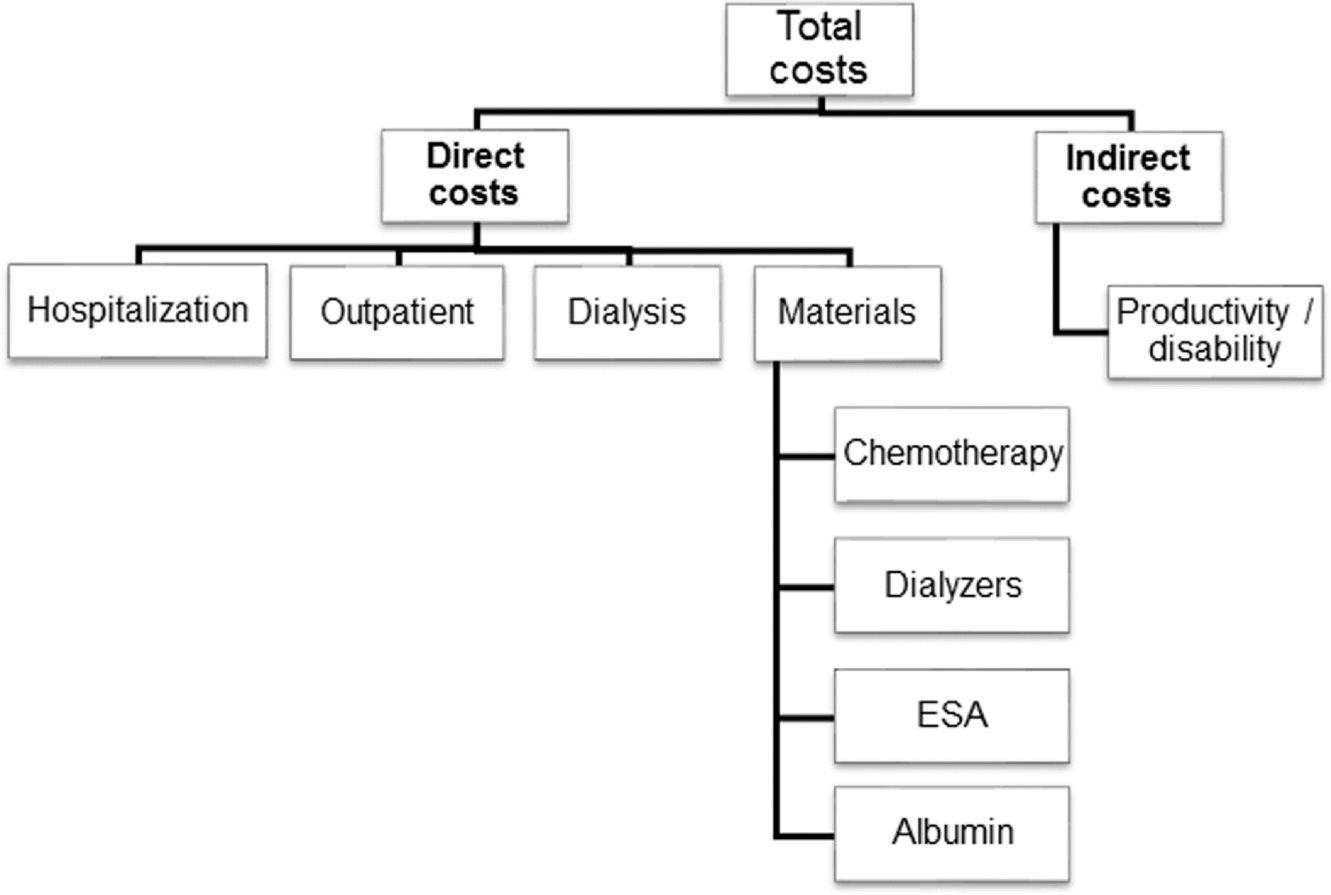
Cost analysis structure.

For new onset MM, baseline was defined as the date of bone marrow biopsy, whereas for relapsing disease the date of serological recurrence of FLCs was chosen. The primary endpoints were survival at 3, 6 and 12 months after baseline, and the hematological response according to the criteria of the IMWG [21]. The secondary end points were FLC reduction at day 12 and 21; recovery of renal function (defined as stop of renal replacement therapy); glomerular filtration rate (expressed as estimated GFR calculated by MDRD study equation) at 3, 6 and 12 months after baseline, respectively; as well the total costs generated by the respective treatment for MM (Fig 2).

### Chemotherapy regimens

Induction chemotherapy consisted of bortezomib in a dose of 1.3 mg/m^2^ once weekly on days 1, 8, 15, 22 in a cycle of 5 weeks, high dose dexamethasone, 10 to 40 mg daily on days 1, 8, 15, 22 in a cycle of 4 weeks in all patients. In some patients, the following chemotherapeutic agents were used in addition to bortezomib/dexamethasone for induction and/or maintenance chemotherapy: cyclophosphamide (HCO: 3, Control: 1), thalidomide (HCO: 3, Control: 2), lenalidomide (HCO: 3, Control: 4), doxorubicin (HCO: 0, Control: 3), vincristine (HCO: 0, Control: 2), melphalan (HCO: 5, Control: 2), carmustine (HCO: 0, Control: 1) and bendamustine (HCO: 0, Control: 1). Treatment of patients with relapsing MM was performed with bortezomib/dexamethasone (VD), lenalidomide/dexamethasone (Rd) or bortezomib/thalidomide/dexamethasone (VTD). Six patients younger than 65 years underwent autologous stem cell transplantation (ASCT) including conditioning with melphalan.

### Laboratory measurements

Serum FLC concentrations were measured with the FREELITE® immunoassay (The Binding Site, Birmingham) immediately before and after dialysis sessions on a daily basis after initiation of renal replacement therapy and thereafter at days 12 and 21. In four patients belonging to the conventional treatment group and having been treated before regular use of FLC measurements was standard, no serum FLC measurements were available.

### Dialysis treatment

Patients in the HCO group were dialyzed according to the schedule by Hutchison et al. [14]. We used the same protein leaking dialyzer (Gambro HCO 1100 Theralite™) in postdilution mode for single use only, and switched to the larger HCO 2100 Theralite™ filter when it became available in 2010. HDF was performed over six hours daily for the first five days, followed by alternate days over the following 12 days. Afterwards, patients were treated four hours thrice weekly until sustained reduction of FLC blood levels was reached (< 500 mg/l; measured after the long interdialytic interval). Albumin was not substituted routinely.

### Cost analysis

Direct costs included hospitalizations, outpatient follow-up, dialysis treatment and materials as billed according to official tariffs in Switzerland before 2012 and the implementation of DRG’s. Hospitalization costs were evaluated from the number of hospital days multiplied by costs per hospital day. The latter were determined by two different methods: First, approximate computation using patient data of 5 patients from the Stadtspital Waid Zurich (data of other patients not eligible); second, from data on file of the Swiss Federal Institute of Statistics (BFS, Bundesamt für Statistik) regarding inpatient hospital costs in the year 2011 for patients with multiple myeloma and renal failure. As both approaches concurred well, we based our analysis on the data of the BFS. Outpatient costs were calculated from the average number of outpatient controls per day of illness, multiplied by number of total illness days and the median cost per outpatient control. For the latter, again, approximate computation from data of 5 patients of the Stadtspital Waid Zurich was used: Median costs per outpatient visits and average number of outpatient visits per day of illness were calculated. Dialysis tariffs are uniform throughout Switzerland and treatments were reimbursed with CHF 490 per session until 2012 and with CHF 530 thereafter. In our analysis, dialysis costs without filters were calculated based on these tariffs and after subtraction of CHF 30 for the average price of conventional HD membranes. Indirect costs for loss of productivity were calculated from the average gross domestic product (GDP) per patient and per day of disability. Patients were excluded from this analysis if or as soon as they were retired. GDP was determined for the time period 2005 through 2013 [22].

### Statistical analysis

All results are given as means±standard deviations unless stated otherwise. Univariate comparisons for differences in continuous variables between treatment groups have been performed by Student’s T-test. Data without normal distribution were analyzed by Mann-Whitney-U test. For comparisons of grouped variables with ordinal outcomes a Chi-Square test was employed. Differences in survival and time to recovery from renal failure were assessed by Cox regression analysis with death and freedom of renal failure, respectively, as dependent variables, and type of dialyzer membrane as independent variable. Due to the low number of patients in the analysis, only age has been used as covariate for Cox regression. P-values ≤ 0.05 were chosen for statistical significance. All analyses were performed with SPSS statistics for Windows, release 20.0 (IBM corporation).

The collection, analysis and publication of data for this study have been approved by the ethics committee of the Canton of Zurich, Switzerland (Kantonale Ethikkommission Zürich). All patient records were de-identified and analyzed anonymously.

## Results

Treatment groups were comparable regarding age at diagnosis, gender, BMI, comorbidity index, eGFR at baseline and insurance modality (Table 1). In 14 patients, *de novo* MM was diagnosed, 5 patients had relapsing MM. The majority of study patients (N = 15) were stage IIIB, the remaining stage IIB. FLC types and concentrations are given in Tables 2 and 3, respectively.

**Table 1:** Demographic baseline characteristics among treatment groups.

	HCO (N = 12)	Control (N = 7)	P-value
Age, years	62.5 ± 13	63.9 ± 11	0.823
Male sex, n (%)	10 (83)	4 (57)	--
BMI, kg/m^2^	24.8 ± 3	23.7 ± 4	0.538
Comorbidity index (Charlson score)	4.8 ± 1.9	4.2 ± 0.4	0.384
eGFR at baseline, ml/min/1.73m^2^	21 ± 21	31 ± 40	0.504
Complimentary health insurance, n (%)	2 (17)	2 (29)	0.565
Newly diagnosed MM, n (%)	10 (83)	4 (57)	0.452

**Table 2:** Distribution of free light chain (FLC) types among treatment groups.

Type of MM	HCO	Control
Free-κ	4	1
IgG-κ	2	0
Free-λ	2	3
IgG-λ	3	2
IgA-λ	1	1

**Table 3:** Free light chain (FLC) serum concentrations among treatment groups.

	HCO	Control	P-value
Baseline (BL), mg/l	11’924 ± 12‘887	8’043 ± 4’916	0.625
Maximum, mg/l	12’652 ± 12’650	9’743 ± 7’723	0.714
Day 12, mg/l	3’543 ± 2710	4’888 ± 6’437	0.591
Day 21, mg/l	2’678 ± 3042	2’549 ± 3’648	0.950
Reduction on day 12 vs. BL, %	-23.6	-19.4	0.702
Reduction on day 21 vs. BL, %	-16.4	-10.6	0.698

**Fig 3:**
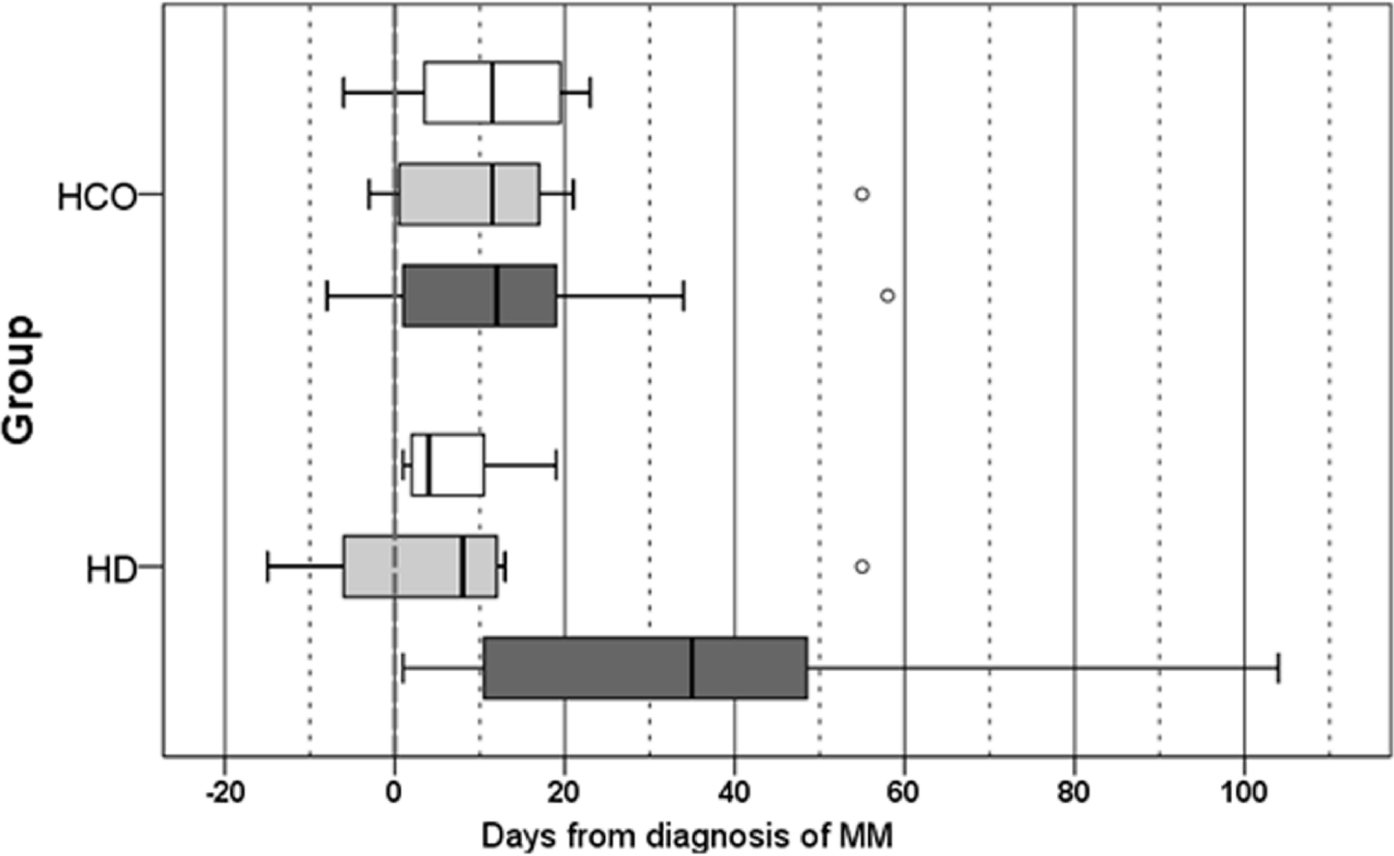
Time intervals from day of clinical MM diagnosis (= 0) to start of chemotherapy, kidney biopsy and renal replacement therapy (median, 25^th^ percentile, 75^th^ percentile, minimum, maximum).

All 19 patients had severe acute kidney injury (Table 4) requiring dialysis either at first presentation (HCO: 6; Control: 2) or during follow-up (HCO: 6; Control: 5). Histological diagnosis of cast nephropathy could be confirmed in all patients. Time intervals from diagnosis of MM to start of chemotherapy, renal biopsy and first dialysis did not differ significantly between groups (Table 5; Fig 3). The median cumulative dose of bortezomib per patient was not different between groups with 40 and 38 mg in the HCO and the control group, respectively. Six patients qualified for ASCT. Time from kidney biopsy to renal replacement therapy was clearly shorter in the HCO group with a median of only 0.5 days versus 35 days in the control group, mainly because in many patients of the former group dialysis was started before kidney biopsy had been available. Time from hematological diagnosis of MM to end of follow-up was comparable between the HCO and control group with 816±729 and 796±456 days, respectively.

**Table 4:** Course of renal function from time of 1^st^ renal replacement therapy session until month 12 thereafter.

	eGFR BL		eGFR Mo 3		eGFR Mo 6		eGFR Mo 12	
	Mean	CI (95%)	Mean	CI (95%)	Mean	CI (95%)	Mean	CI (95%)
HCO	7.7 ± 3	5.7–9.8	30 ± 26	12.5–47	33 ± 28	14.2–52	40 ± 27	20–61
Control	7.4 ± 3	4.8–9.9	16 ± 10	6.7–25	17 ± 11	5.3–28	21 ± 11	9.4–32

**Table 5:** Relevant time intervals (mean±SD) for diagnostic and therapeutic measures among treatment groups.

Time from… / to… (days)	HCO	Control	P-value
Diagnosis / Start of chemotherapy	13 ± 8	7 ± 7	0.133
Diagnosis / Renal biopsy	12 ± 16	10 ± 22	0.833
Diagnosis / First dialysis	14 ± 18	37 ± 35	0.147
Diagnosis / Death or End of follow-up	816 ± 796	796 ± 456	0.943
First dialysis / Last dialysis	191 ± 294	536 ± 478	0.119
Last dialysis / Death or End of Follow up	681 ± 762	223 ± 348	0.155

**Fig 4:**
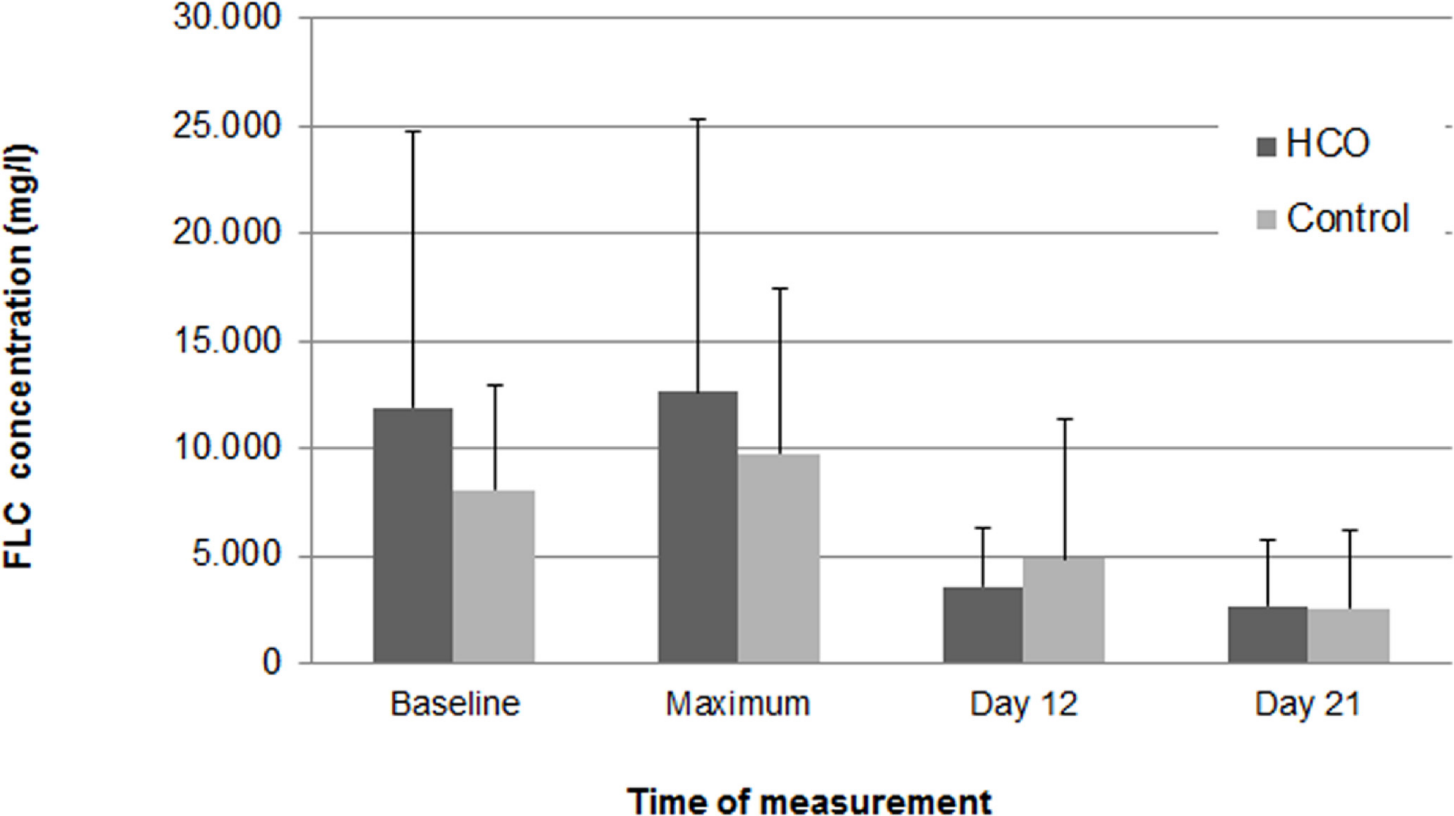
Serum FLC reduction from baseline to day 12 and 21. Serum FLC reduction from baseline to day 12 and 21 was 23.6% (p = 0.029) and 16.4% (p = 0.024), respectively, in the HCO group versus 19.4% (p = 0.057) and 10.6% (p = 0.047), respectively, in the control group. The differences between treatment groups at both 12 and 21 days were not statistically significant.

The percentage FLC reduction at day 12 and 21 was 23.6 and 16.4, respectively, in the HCO group versus 19.4 and 10.6, respectively, in the control group (p = NS; Fig 4). In the HCO group 11 out of 12, and 10 out of 12 patients had a reduction in their FLCs at 12 and 21 days, respectively. Among those, 50% and 30% of patients had an FLC reduction beyond 25% at 12 and 21 days, respectively. Of the 3 individuals for which FLC determinations were performed in the control group, all showed a reduction both at 12 and 21 days of treatment. However, only 1 out of 3 patients at 12 days, but none after 21 days had a reduction beyond 25% from baseline. With regard to hematological response, the percentage of patients with complete response (CR) was 17% and 0% in the HCO and control group, respectively; 33% and 43% for partial response (PR); 33% and 29% for very good partial response (VGPR); and 8% and 14% for progressive disease. One patient in the HCO group could not be classified, whereas one patient in the control group had stable disease (Table 6).

**Table 6:** Tabulation of all subjects for disease classification (Salmon-Durie), hematologic response, and additional chemotherapeutic agents used according to treatment group.

	Benda	Carmu	Doxo	Vincr	Lena	Thali	Cyclo	Melph	Stadium	Hematol Response
**High Cut-Off**	0	0	0	0	0	1	0	0	IIIB	Progressive
	0	0	0	0	0	0	0	1	IIIB	VGPR
	0	0	0	0	0	1	0	1	IIIB	VGPR
	0	0	0	0	0	0	0	0	IIIB	VGPR
	0	0	0	0	1	0	0	0	IIIB	PR
	0	0	0	0	0	0	1	1	IIIB	CR
	0	0	0	0	0	1	0	0	IIB	PR
	0	0	0	0	0	0	1	1	IIIB	PR
	0	0	0	0	0	0	0	0	IIIB	Inconclusive
	0	0	0	0	1	0	0	0	IIB	CR
	0	0	0	0	1	0	1	0	IIIB	PR
	0	0	0	0	0	0	0	1	IIIB	VGPR
**Control**	0	1	1	1	1	0	1	1	IIIB	PR
	0	0	0	0	0	0	0	0	IIB	PR
	0	0	1	0	1	0	0	0	IIIB	PR
	1	0	1	1	0	1	0	1	IIIB	Stable
	0	0	0	0	0	0	0	0	IIB	Progressive
	0	0	0	0	1	0	0	1	IIIB	VGPR
	0	0	0	0	1	1	0	0	IIIB	VGPR

Benda = Bendamustine; Carmu = Carmustine; Doxo = Doxorubicin; Vincr = Vincristine; Lena = Lenalidomide; Thali = Thalidomide; Cyclo = Cyclophosphamide; Melph = Melphalan. 0 = No therapy; 1 = Therapy with respective agent received

**Fig 5:**
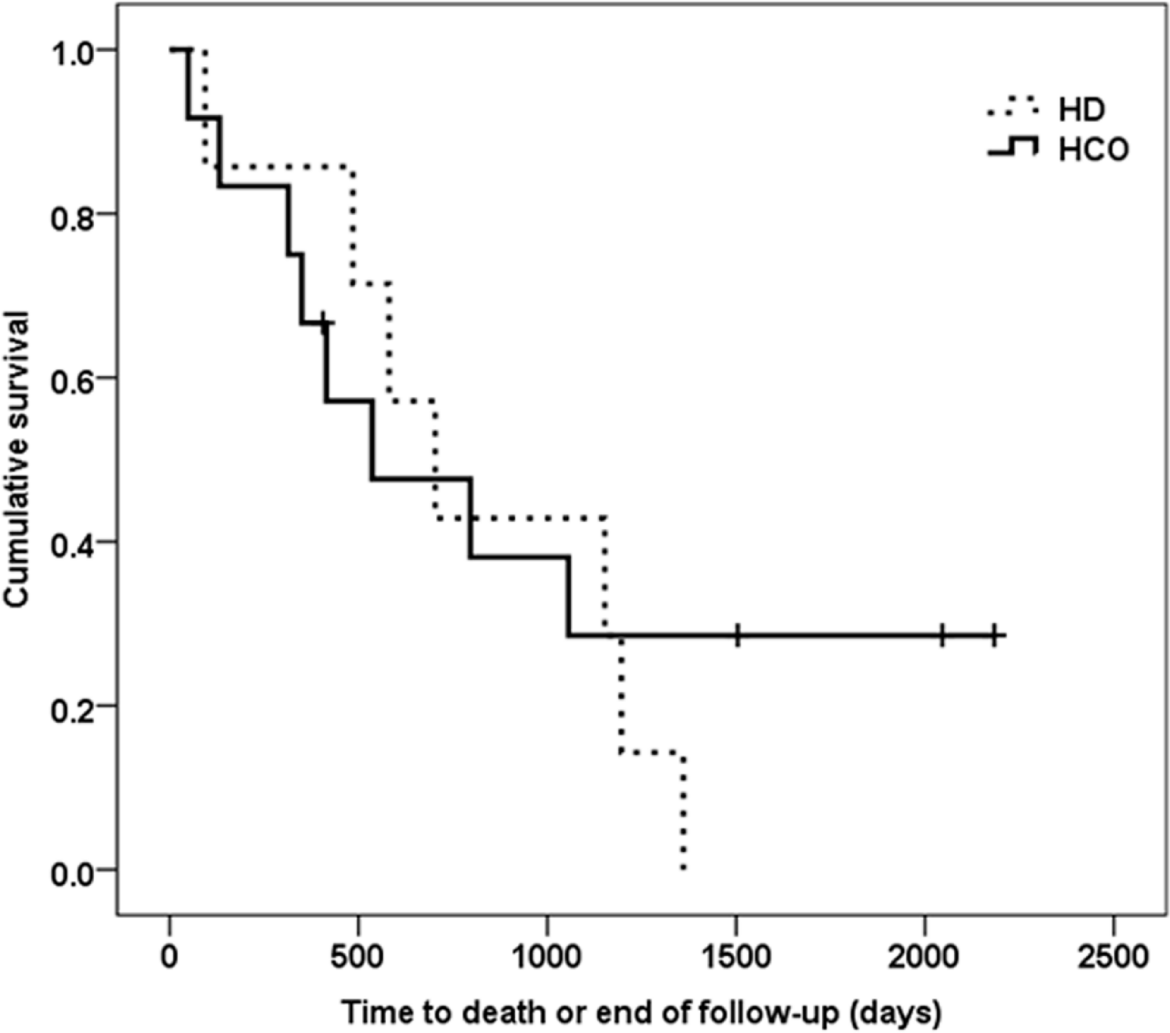
Cumulative patient survival. Kaplan-Meier curve depicting cumulative patient survival censored for death. The solid line represents patients treated with HCO membranes, the dashed line represents patients treated by conventional HD filters. Cox regression analysis was performed with survival as dependent and treatment group (HCO, control) and age as independent variables. P-value for difference between groups was 0.815.

By the end of follow-up (December 31 2015), 4 patients treated with HCO dialyzers were still alive, while all patients in the control group had died (Fig 5). However, this difference was not statistically significant by Cox regression analysis with adjustment for age (p = 0.815). Survival at 3, 6 and 12 months was 92%, 83%, and 75%, respectively, in the HCO group, versus 86% at all time intervals in the control group. Of note, patients who have died had significantly less HCO sessions and were older at time of diagnosis than patients who survived (p = 0.015 and 0.025, respectively). Furthermore, renal function of patients who have died during the study period tended to be worse both at 6 and 12 months after diagnosis of MM (p = 0.145 and p = 0.149, respectively).

**Fig 6:**
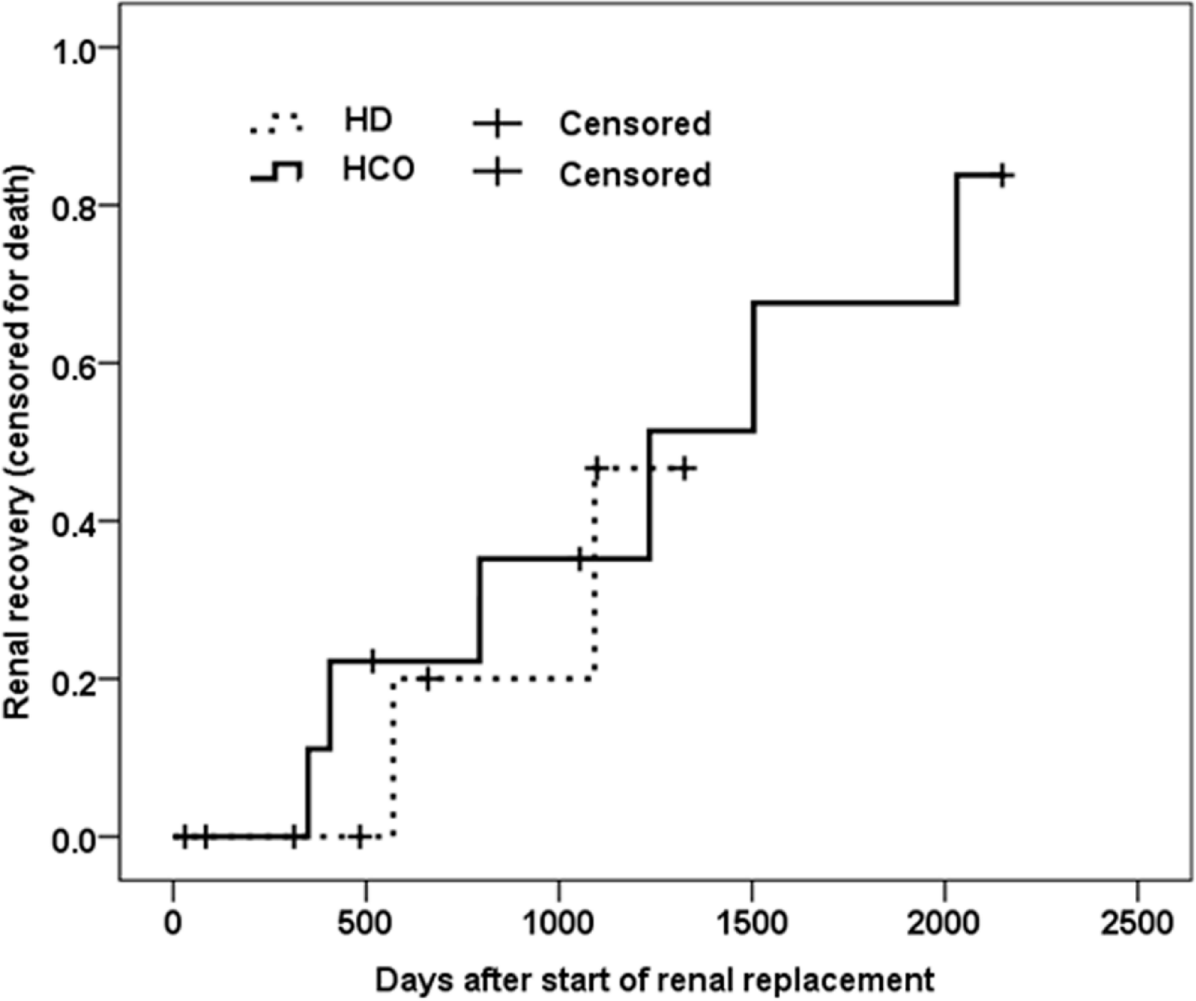
Renal recovery. Kaplan-Meier curve depicting renal recovery, defined by independence from dialysis, censored for death and end of follow-up. The solid line represents patients treated with HCO membranes, the dashed line represents patients treated by conventional HD filters. Cox regression analysis was performed with renal recovery as dependent and treatments group (HCO, HD) and age as independent variables. P-value for difference between groups was 0.246.

A non-significant trend to better renal recovery was seen in patients treated with HCO dialyzers versus controls (p = 0.246; Fig 6). At approximately 6 months after initiation of dialysis, 6 out of 12 patients in the HCO group were independent of HD. In contrast, the shortest time to renal recovery in the control group was approximately 7 months. Moreover, trends towards better renal function at 12 months after diagnosis of MM as well as lesser time on renal replacement therapy can be noted for HCO versus control (p = 0.109, and 0.066, respectively). Furthermore, eGFR levels achieved in patients with renal recovery were clearly better in the HCO versus the control group (Table 4). Complete renal response rate was achieved in 10.5% and 0% of HCO and control patients, respectively, partial renal response in 15.8% and 5.3%, and minor renal response in 26.3% and 15.8%. In both groups, no correlation was found between renal recovery and degree of FLC reduction or duration of dialysis therapy.

**Fig 7:**
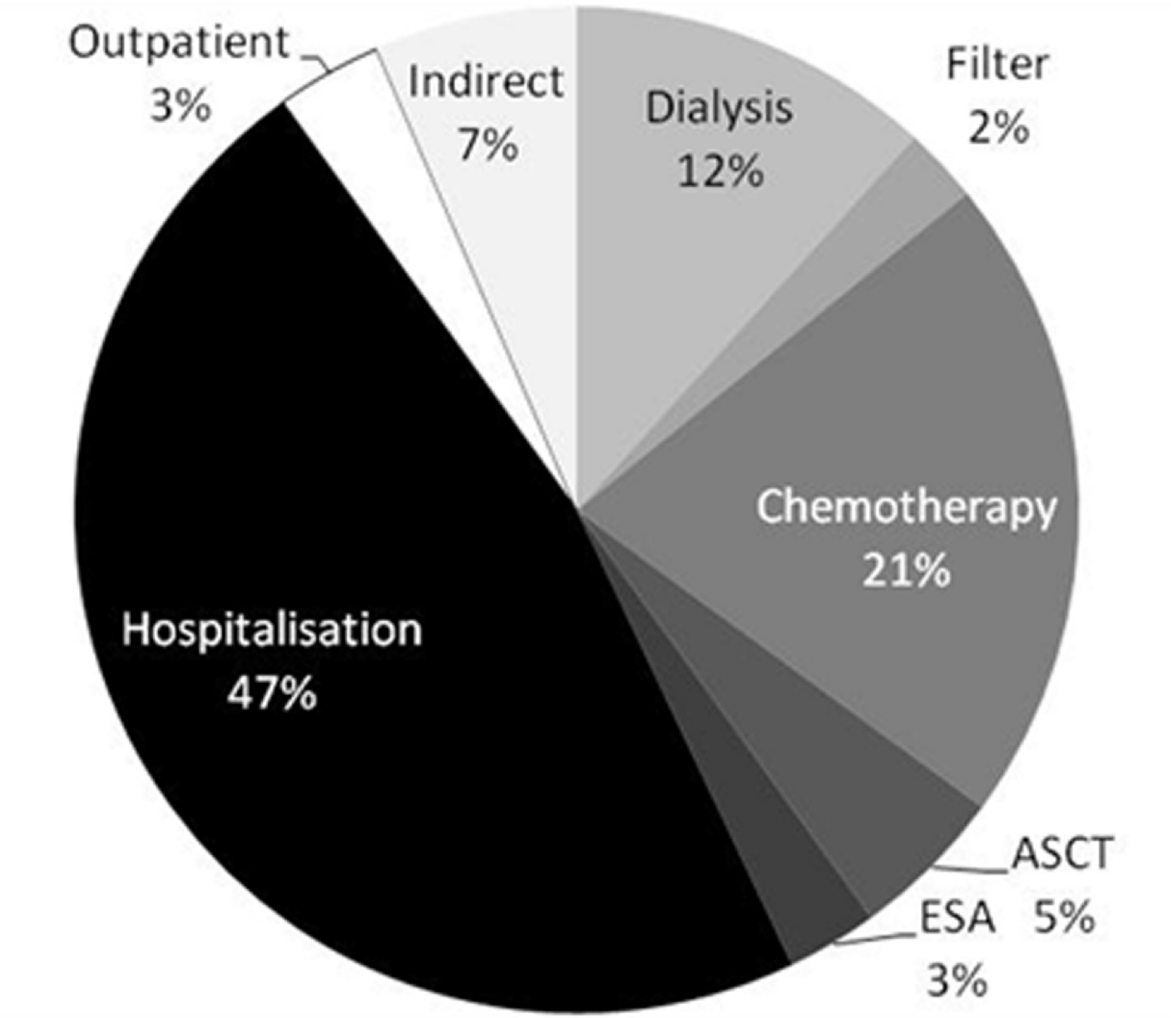
Percentage distribution of total costs in the entire study population.

Economic analysis revealed that, regardless of dialysis treatment allocation, hospitalization costs made up for almost half of total costs (47% and 48% in the HCO versus control group, respectively. Fig 7). Chemotherapy accounted for 21% of total costs (HCO: 19%, control: 24%). Dialysis treatment, excluding filters, was the third largest cost factor with 12% (HCO: 15%, control: 7%). This was followed by indirect costs for loss of productivity and disability with 7% (HCO: 6%, control: 8%). ASCT made up for 5% of total expenditures (HCO: 5%, control: 4%). Finally, costs for dialyzers (HCO: 2%, control: 1%), outpatient treatment (HCO: 4%, control: 3%) and erythropoietin (HCO: 2%, control: 5%) had minor economic impact. Reimbursement for albumin substitution accounted for less than 1% and was, therefore, neglected. Costs for dialyzers were about three times higher in the HCO versus the control group (p = 0.002). However, all other costs as well as total expenditures did not significantly differ between groups (Table 7).

**Table 7:** Analysis of median direct and indirect treatment costs per patient among treatment groups.

Cost (CHF)	HCO	Control	P
Dialysis	18’170	19’320	1.000
Filter	8’286	3’829	0.002
Chemotherapy	33’671	36’942	0.673
ASCT	13’333	11’429	0.834
ESA	2’835	10’087	0.261
Hospitalization	129’285	157’170	0.672
Outpatient	4’845	7’163	0.447
Indirect	14’119	20’789	0.807
Total	230‘056	222‘864	1.000
Total/day	381	340	0.902

## Discussion

This small retrospective study shows a clear trend to better survival and to faster and better renal recovery from cast nephropathy in patients treated with HCO versus conventional dialyzers. Both patient survival and renal recovery were significantly correlated with the extent of serum FLC reduction in the HCO group. Finally, cost analysis revealed an overall neutral effect for renal replacement therapy with HCO membranes. To the best of our knowledge, this is the first study comparing outcomes and costs in patients with MM and renal failure treated with HCO membranes in comparison to conventional hemodialysis.

Our study supports previous publications suggesting high FLC clearance with HCO membranes by demonstrating a reduction in serum free light chains of about 80% (Fig 4). This compares favorably with the findings of Hutchison et al. (JASN, 2009), who reported a lowering between a minimum of 50% to a maximum of 97% with a mean of 82% in 15 patients accomplishing treatment over the assigned period and having uninterrupted chemotherapy throughout. With regard to survival as the primary end point of our analysis no statistically significant difference was detectable between groups. However, a clear trend to better survival in patients treated with HCO versus conventional hemodialysis membranes was shown (Fig 5). Whereas no patient in the control group survived after a median follow-up of 703 days (range: 94–1360), 4 out of 12 patients in the HCO arm were still alive after a median follow-up of 1775 days (range: 1249–2183). Reduction in serum FLC was by about 25% higher in survivors versus non-survivors both at days 12 and 21 of treatment. Moreover, comparing the 15 individuals with FLC levels available at day 21, all patients with a reduction in FLC versus only 1 of 3 patients without reduction were still alive after 12 months (p = 0.029). Overall, 1-year patient survival in the HCO group was with 75% identical to that in Hutchison’s case series published 2009 [14]. Finally, in our own series, patients living at the end of follow-up were significantly younger (51±16 vs. 66±9 years, p = 0.025) and reached independence of renal replacement therapy earlier compared to non-survivors (after 92±53 vs. 378±429 days, p = 0.210). Again, these results are in accord with previous studies demonstrating better survival in MM patients with cast nephropathy becoming free of dialysis [14, 23, 24].

Clear trends for faster and better renal recovery were found for patients treated with HCO membranes in the present investigation (Fig 6, Table 5). After 3 months already, 5 of 12 HCO vs. only 1 of 6 control patients no longer required renal replacement. Individuals free of dialysis at month 3 had significantly higher baseline serum FLC concentrations (18’800±15’300 vs. 6’000±4’200 μg/l; p = 0.032), but also a clearly higher absolute FLC reduction at 21 days of treatment (16’200±13’200 vs. only 4’900±5’100 μg/l, p = 0.035). Moreover, patients having recovered renal function at month 3 had started dialysis earlier at a median 12 vs. 35 days after diagnosis of MM (p = 0.048), although with similar eGFR of 7 vs. 8 ml/min/1.73m^2^. Median eGFR of those patients being free of treatment after 3 months was 46 ml/min/1.73m^2^ (range: 22–91) in the HCO group (N = 5) versus 37 ml/min/1.73m^2^ in the only patient from the control group. Again, our findings are in line with those by Hutchison and colleagues, who reported renal recovery in 65% of their study patients at month 3 of treatment with HCO membranes [14], and renal function being at an eGFR of 40 ml/min/1.73m^2^ (range 11–83). Similar results for HCO membranes were published by Peters in 2011 [18] and Zanetti in 2015 [17]. After month 3 to end of follow-up of our study, 2 more patients in the HCO group recovered after 136 and 139 days of dialysis, in contrast to only one more individual in the control group after 229 days. These results are clearly superior to that published previously for MM patients with cast nephropathy being treated by conventional hemodialysis with renal recovery rates in the range of 0% to 37% only, whereas the use of HCO membranes in our own study and the one by Hutchison et al. resulted in freedom from dialysis in 58% and 74%, respectively.

Standard use of HCO filters has been prevented, so far, due to cost restrictions. Filter cost was the only factor in our economical analysis that differs significantly between groups (Table 7). Of note, expenses for dialysis filters in the HCO group, including the average of 13±12 HCO membranes, were only about 3 times higher, because patients in the control group were substantially longer dialysis dependent and required 132±103 conventional dialyzer membranes (HCO patients: 65±105). More importantly, other direct and indirect treatment costs for in- and outpatient medical services were lower in the HCO vs. control group. This is explained by shorter hospitalization of a median 76 versus 93 days in HCO and control patients, respectively (p = NS). Consequently, total expenditures were comparable between groups.

Our investigation has several limitations. First of all, it has been conducted retrospectively, and, therefore, is prone to selection bias. This is particularly relevant with regard to comparable patient populations. Although groups were fairly similar for age, sex, comorbidity, baseline renal function and new onset myeloma (Table 1), they were nevertheless treated in different institutions and years. This, theoretically, may have impacted on other therapy aspects too, such as general medical care and medication. However, we took great care in defining stringent inclusion and exclusion criteria for patient selection into the study. Most importantly, all patients received identical standard of care regarding chemotherapy with dexamethasone and bortezomib. Moreover, all patients had biopsy proven cast nephropathy and comparable hematological characteristics in order to ensure similar disease outsets. Also, the better complete hematological response in the HCO group did not reach statistical significance. Nevertheless, it cannot be ruled out completely that the better outcomes both for survival and renal recovery were mainly due to a better effect of chemotherapy in the HCO group. A second limitation applies to the rather small number of patients included in our analysis, as cast nephropathy with renal failure is a rather rare disorder. Consequently, we were unable to identify more patients fulfilling our strict inclusion criteria even after a nationwide search among the Swiss nephrology community. Not surprisingly, all the published series on this topic have about the same size, as, for example, the study by Hutchison et al. with a total of 20 patients [14]. Unfortunately, as a consequence, most of our analyses did not reach statistical significance despite clear trends for positive treatment effects. Better evidence is needed and can be expected from prospective studies such as the EuLite (ISRCTN45967602) and the MYRE (NCT01208818) trials. Finally, our cost analysis of cast nephropathy treatment may not be applicable to other countries with different reimbursement policies.

In summary and conclusion, this is the first study comparing the use of High Cut-Off versus conventional dialysis membranes in patients with renal failure from cast nephropathy regarding clinical endpoints and therapy costs. In the context of previous studies using HCO hemodialysis therapy in a comparable setting our findings clearly suggest a benefit for HCO membranes with regard to improved patient survival, renal recovery and renal function in patients with myeloma associated renal failure. In the absence of results from randomized prospective studies on this topic and given neutral costs compared with conventional hemodialysis treatment the use of HCO membranes in patients with dialysis dependent cast nephropathy may be recommended under stringent conditions. In order to reach definite conclusions prospective controlled trials are required.
